# Syphilitic Retraction of the Muscles

**Published:** 1843-04

**Authors:** 


*Syphilitic Retraction of the Muscles.*—This is a disease of rare occurrence, and which has only of late received attention. It affects most frequently the flexor muscles of the fore-arm, if we may be allowed to form an opinion from the generality of cases observed at the venereal hospital, under Ricord. The three patients who presented this remarkable affection had arrived at that point of constitutional infection characterized by the symptoms which are denominated tertiary by Ricord. In all these the retraction was very similar; the flexors of the fore-arm being affected by it. The muscles appeared shortened, as a result of the permanent contraction, which did not permit the extension of the fore-arm; but their tissue, though firm, presented no appreciable alteration. An important symptom was the peculiar pain which existed in the contracted part; this pain was increased at night, and resembled closely that experienced in syphilitic affections of the bones. In one of the patients the retraction was cotemporary with tertiary ulcerations of the throat; in another, with periostitis of the tibia. These patients were submitted to the treatment of iodide of potassium. The success, under its influence, was as prompt and easily obtained as in other tertiary symptoms. The pains ceased in each one as soon as the fifth or sixth day. The movements of the limbs underwent a progressive amelioration, and were soon perfectly restored.—*Lond. Med. Gaz.*, July, 1842, from *Bull. de Thérapeut.*

